# The impact of a national exercise intervention on lifestyle habits among individuals with schizophrenia spectrum disorders: results from the FitForLife intervention study

**DOI:** 10.1186/s13104-026-07801-x

**Published:** 2026-04-15

**Authors:** Amanda Cassel Pegelow, Maria Skott, Örjan Ekblom, Catharina Lavebratt, Yvonne Forsell

**Affiliations:** 1 Department of Global Public Health, Karolinska Institutet, Stockholm, Sweden; 2 Department of Clinical Neuroscience, Karolinska Institutet, Stockholm, Sweden; 3 Department of Physical Activity and Health, The Swedish School of Sport and Health Sciences (GIH), Stockholm, Sweden; 4 Department of Molecular Medicine and Surgery, Karolinska Institutet, Stockholm, Sweden; 5 Center for Molecular Medicine, Karolinska University Hospital, Stockholm, Sweden

**Keywords:** Alcohol, Dietary habits, Lifestyle habits, Physical activity, Schizophrenia spectrum disorders, Smoking, Nicotine

## Abstract

**Objectives:**

Individuals with schizophrenia spectrum disorders face challenges in maintaining healthy lifestyle habits and have a significantly reduced life expectancy, partly due to increased risks of somatic illness. While physical activity is known to offer broad health benefits, its influence on other lifestyle behaviors—such as diet, alcohol consumption and nicotine use—remains unclear. This study aimed to investigate whether an exercise intervention could affect these lifestyle habits in individuals with schizophrenia spectrum disorders (F20–29). This sub-study was part of a single-arm intervention conducted across 16 outpatient units in Sweden. Participants completed questionnaires before and after the six-month intervention. Analyses included descriptive comparisons, non-parametric tests and ordinal logistic regressions.

**Results:**

In total, 149 individuals participated. After the intervention, nicotine use showed minimal change. There were patterns of decreased alcohol use and decreased fruit, vegetable and meat consumption, along with increased candy consumption and binge drinking. Further interventions are needed to promote healthy lifestyle habits among individuals with schizophrenia spectrum disorders.

*Trial registration* The trial is registered as “Fitforlife- Exercise in Care of Psychosis” in the ClinicalTrials.Gov with the ID number: NCT04239612 (2019–10–28).

**Supplementary Information:**

The online version contains supplementary material available at 10.1186/s13104-026-07801-x.

## Introduction

Individuals with schizophrenia spectrum disorders (SSD) experience significantly reduced life expectancy (12–15 years) and elevated risks of somatic illnesses [1]. The shortened life expectancy is attributed to both medication side effects and unhealthy lifestyle habits, which contribute to somatic diseases that are partly preventable such as cardiovascular and metabolic disease [2–5].

Individuals with SSD exhibit significantly lower levels of physical activity and higher levels of sedentary behavior compared to the general population [6–8]. Increased physical activity has numerous benefits, including enhanced cognitive functioning, improved psychosocial functioning, and cardiovascular advantages [7–10]. In this sub-study of the FitForLife project, the aim was to investigate whether a six-month physical exercise intervention impacted other lifestyle habits such as nicotine use, alcohol use or dietary habits in individuals with SSD.

Nicotine use is common in individuals with SSD and is a major risk factor for cardiovascular and respiratory diseases [11–13]. Alcohol use has been associated with increased positive symptoms and worse psychosocial functioning, as well as with risks of multiple non-communicable diseases [14, 15]. Poor dietary patterns are common in the studied population [16, 17], contributing to significantly higher rates of obesity, metabolic syndrome and likely contributing to the different intestinal microbiome [6, 9, 18]. These lifestyle habits contribute to the shorter life expectancy in this group [11–14, 19].

Although medication and psychological therapy remain central to the treatment of SSD, current approaches often overlook the role of lifestyle interventions, despite their importance. Building upon the benefits of increased physical activity, a key question is whether physical exercise interventions could also improve other lifestyle habits, offering a comprehensive and cost-effective strategy for individuals with SSD [20, 21].

## Methods

### Study design and population

This study employed a quantitative sub-study design nested within a single-arm intervention: FitForLife [22, 23]. The design was selected to ensure that all eligible patients could access the intervention. It was implemented at 16 psychiatric outpatient units specialized in care of psychosis in Sweden. The intervention consisted of at least one group exercise session per week led by patients educated for the task. The exercise intervention was included as a part of the individual care plan for the patients and was followed up at all meetings with the patients. Additionally, the participants were encouraged to contact and remind each other before the sessions. The unit also had posters about the sessions in the waiting rooms. The implementation occurred between 2021 and 2023.

Participants were adults (≥ 18 years) with an SSD diagnosis receiving care at one of the 16 units. Each potential participant was clinically assessed by their care provider that decided if the patient was eligible. Exclusion criteria included conditions preventing participation in the exercise sessions, such as acute psychosis or somatic illnesses where physical exercise was contraindicated. For this sub-study, individuals who did not answer the lifestyle-related questions were excluded. This study adheres to STROBE guidelines.

### Data collection and measures

Recruitment was conducted at the outpatient units, with all eligible patients asked to participate. Data were collected at baseline and at six-month follow-up via (i) questionnaires, (ii) examinations (e.g., bodily measurements such as height and weight), (iii) interviews about general background information (e.g., age, sex, education and living situation), (iv) a fitness test and (v) medical records (the first F-diagnosis recorded, duration of the diagnosis and antipsychotic medication), all collected/recorded by qualified staff at the outpatient units. Questionnaires were completed either on paper or through a mobile app according to participants preference. Written informed consent was obtained at baseline.

The questionnaire items relevant to this sub-study covered alcohol use, nicotine use, and dietary habits. Illicit drug use was a potential research question. However, no participants reported using illicit drugs. Nicotine use was assessed with a yes/no question. The alcohol-related questions were drawn from the widely used Alcohol Use Disorders Identification Test (AUDIT) [24]. Dietary habits were measured by asking how often the participants had consumed fruit, vegetables, meat, and candy/soda in the past week, with response categories ranging from “1–2 times/week” to “6 or more times/week”. The questionnaire is provided as a supplementary file. During the study period, session attendance was recorded and included in the patient medical records. At the six-month follow-up, the same information was collected as at baseline.

### Data analysis

The statistical analyses were conducted using STATA 18. The proportions of change within each variable between pre- and post-assessments were calculated, and two-sided non-parametric tests (Fisher’s exact test and Wilcoxon signed-rank test) were conducted.

Further analyses were conducted with lifestyle habit categories being dependent variables in ordinal logistic regression models. The ordinal logistic regression models adjusted for age, sex, education, living situation, and BMI to control for potential confounders. The significance level was set at α = 0.05 in all analyses. The regression results were reported as odds ratios (OR) with 95% confidence intervals (CI). A binary logistic regression was used to examine whether individuals lost to follow-up differed systematically from respondents regarding the covariates.

Additionally, an interaction analysis was conducted to assess whether the frequency of physical activity sessions modified the effect of the intervention. This did not show any differential results and was therefore not included in the results section.

## Results

### Sociodemographics

A total of 149 individuals were recruited, of whom 52% were men, 37% had an F20 (schizophrenia) diagnosis, 22% lived together with another adult, and the mean age was 47.5 years (Table 1). Participants attended an average of 19.3 exercise sessions, with a standard deviation of 13. A total of 44 participants (29.5%) did not complete any follow-up questions. Each analysis specifies the sample size (N) based on valid responses. No significant differences were identified between respondents and non-respondents for the covariates tested.

**Table 1:** Baseline characteristics

	Mean N (%)
Total	149
Sex	
Male	74 (51.7%)
Female	69 (48.3%)
Age	47.5
20–29	6 (4.2%)
30–39	32 (22.4%)
40–49	44 (30.8%)
50–59	41 (28.7%)
60–69	17 (11.9%)
70–75	3 (2.1%)
F-diagnoses	
20 Schizophrenia	51 (37.0%)
22 Persistent delusional disorder	10 (7.2%)
23 Acute and transient psychotic disorder	11 (8.0%)
24 Induced delusional disorder	1 (0.7%)
25 Schizoaffective disorders	21 (15.2%)
28 Other nonorganic psychotic disorders	1 (0.7%)
29 Unspecified nonorganic psychosis	21 (15.2%)
30–39 Mood affective disorders	7 (5.1%)
Other	15 (10.9%)
Years with diagnosis	13.4
1–5	13 (11.2%)
6–10	39 (33.6%)
11–15	25 (21.6%)
16–20	22 (19.0%)
21–25	7 (6.0%)
26+	10 (8.6%)
Antipsychotics	
No	15 (11.0%)
Yes	121 (89.0%)
Education	
Primary school	28 (23.3%)
Highschool	66 (55%)
Higher education	26 (21.7%)
Cohabitant	
No	97 (78.2%)
Yes	27 (21.8%)
BMI (kg/m^2)	30.4
Normal weight	23 (17.0%)
Overweight	45 (33.3%)
Obese	67 (49.6%)
Do you use nicotine products?	
No	73 (59.8%)
Yes	49 (40.2%)
Alcohol use: AUDIT result	
Abstainer	30 (32.6%)
Low-risk	58 (63.0%)
Harmful/hazardous	4 (4.3%)
Dependence	0 (0%)
How often do you drink alcohol?	
Never	30 (29.1%)
1/month	46 (44.7%)
2–4/month	19 (18.4%)
2–3/week	6 (5.8%)
4+/week	2 (1.9%)
How often do you have 6 + drinks on one occasion?	
Never	79 (76.7%)
Less than 1/month	16 (15.5%)
Every month	7 (6.8%)
Every week	1 (1.0%)
Fruit times/week	
1 to 2	33 (27.3%)
3 to 4	28 (23.1%)
5 to 6	15 (12.4%)
6 or more	45 (37.2%)
Vegetables times/week	
1 to 2	32 (27.1%)
3 to 4	22 (18.6%)
5 to 6	22 (18.6%)
6 or more	42 (35.6%)
Meat times/week	
1 to 2	29 (26.1%)
3 to 4	51 (45.9%)
5 to 6	15 (13.5%)
6 or more	16 (14.4%)
Candy or soda times/week	
1 to 2	73 (62.9%)
3 to 4	24 (20.7%)
5 to 6	9 (7.8%)
6 or more	10 (8.6%)

### Nicotine

At baseline, nicotine use was reported by 40.2% of participants (Table 1). There were minimal changes between baseline and follow-up: two participants quit and two initiated use (Table 2). Due to a lack of variation in responses, no further analysis of nicotine use was conducted.

**Table 2:** Changes between baseline and follow-up data. Showing number of individuals, proportions and *p*-value

	*N*	Decrease *N* (%)	No change *N* (%)	Increase *N* (%)	*p*-value
Nicotine use	73	2 (2.7)	69 (94.5)	2 (2.7)	Fishers exact 1.000
Alcohol use AUDIT category	51	9 (17.6)	41 (80.4)	1 (2.0)	*Wilcoxon signed-rank* **0.011**
Alcohol intake frequency	68	14 (20.6)	46 (67.6)	8 (11.8)	0.208
Binge drinking	65	6 (9.2)	51 (78.5)	8 (12.3)	0.600
Dietary habits					
Fruit times/week	80	26 (32.5)	34 (42.5)	20 (25)	0.376
Vegetables times/week	77	22 (28.6)	41 (53.3)	14 (18.2)	0.188
Meat times/week	72	19 (26.4)	38 (52.8)	15 (20.8)	0.634
Candy or soda times/week	71	10 (14.1)	44 (62.0)	17 (23.9)	0.232
BMI category (kg/m^2)	108	9 (8.33)	97 (89.8)	2 (1.85)	**0.034**

Bold indicates *p* < 0.05

### Alcohol

Harmful/hazardous drinking was observed in 4.3% of participants at baseline (Table 1). Non-parametric tests indicated a change in AUDIT classification (three categories) between baseline and follow-up (*p* = 0.011; Table 2). Table 3 presents the regression models, unadjusted and adjusted. The results show a decrease in AUDIT categories and alcohol use frequency, as well as an increase in binge drinking.

**Table 3:** Ordinal logistic regression with dependent at baseline and follow-up, showing OR and 95% CI

Variable (reference category)	Alcohol use, AUDIT category	Alcohol use frequency	Binge drinking	Fruit times/week	Vegetables times/week	Meat times/week	Candy/soda times/week
Unadjusted							
Time (baseline)							
Follow-up	0.54	0.79	1.04	0.84	0.77	0.98	1.25
	[0.24,1.18]	[0.42,1.46]	[0.46,2.34]	[0.48,1.47]	[0.44,1.36]	[0.54,1.79]	[0.66,2.38]
N	102	136	130	160	157	144	142
Adjusted							
Time (baseline)							
Follow-up	0.55	0.80	1.24	0.92	0.83	0.88	1.10
	[0.22,1.31]	[0.42,1.52]	[0.52,2.94]	[0.49,1.72]	[0.45,1.54]	[0.45,1.69]	[0.55,2.20]
Age (Cont.)	0.98	0.99	0.96	**1.08**	**1.05**	**1.04**	1.00
	[0.94,1.03]	[0.96,1.03]	[0.91,1.01]	[1.04,1.12]	[1.01,1.09]	[1.00,1.08]	[0.96,1.04]
Sex (Male)							
Female	2.29	1.74	0.79	**0.31**	0.81	**0.36**	0.96
	[0.90,5.84]	[0.86,3.52]	[0.30,2.06]	[0.15,0.67]	[0.40,1.68]	[0.17,0.76]	[0.43,2.14]
Cohabitant (No)							
Yes	**3.71**	**2.42**	1.05	1.88	1.68	0.94	0.42
	[1.05,13.12]	[1.08,5.44]	[0.33,3.39]	[0.84,4.21]	[0.75,3.79]	[0.42,2.11]	[0.16,1.10]
Education (Primary school)							
Highschool	1.50	1.17	1.071	**3.30**	0.84	1.00	1.30
	[0.48,4.70]	[0.49,2.81]	[0.37,3.11]	[1.38,7.87]	[0.37,1.91]	[0.44,2.31]	[0.51,3.35]
Higher education	0.32	0.43	0.33	**8.59**	1.36	1.31	1.75
	[0.089,1.12]	[0.17,1.13]	[0.08,1.29]	[2.90,25.46]	[0.50,3.68]	[0.49,3.50]	[0.57,5.33]
Body mass index (Cont.)				1.02	0.99	1.03	1.05
				[0.96,1.08]	[0.94,1.05]	[0.97,1.09]	[0.99,1.13]
N	97	128	122	137	135	124	123

Bold indicates *p* < 0.05

### Dietary habits

Table 1 presents baseline results for dietary habits. After the intervention, fruit, vegetables, and meat consumption had decreased compared to baseline, while candy/soda consumption had increased (Tables 2 and 3). BMI had decreased compared to baseline (*p* = 0.034).

## Discussion

This study showed that a physical exercise intervention alone did not significantly affect other lifestyle habits (nicotine use, alcohol use, and dietary habits) in individuals with SSD. This suggests that additional targeted components may be needed.

### Nicotine

There was a high prevalence of nicotine use in this sample, consistent with prior findings among individuals with SSD, both nationally and internationally [1, 25]. By comparison, there is a low prevalence of daily smoking in the general Swedish population [26]. Nicotine use in SSD may partly reflect nicotine’s role in reducing side effects of neuroleptics and its potential role in symptom management and therefore there might be concerns about symptoms worsening with cessation [27]. In line with this, nicotine use showed minimal change at follow-up, suggesting a need for combined programs [1, 13, 27].

### Alcohol

Harmful/hazardous drinking appeared uncommon in this sample, aligning with prior findings among schizophrenia outpatients [28]. This contrasts with broader reports of substance dependence in SSD, as well as with the general Swedish population (4.3% vs. 15.6%) [1, 29]. The former discrepancy may reflect studies that combine alcohol with other substances, as well as outpatient versus inpatient samples. Social and structural factors – such as stigma, exclusion, social withdrawal, and financial constraints – may contribute to the low prevalence of drinking in this group [30].

The results showed a mixed pattern at follow-up, indicating that exercise alone may not alter drinking behavior in a uniform direction. This is notable as previous studies in other populations link higher physical activity to greater alcohol consumption [31, 32]. Larger samples or longer interventions may help determine whether the observed patterns persist.

### Dietary habits

Although poor dietary habits are frequently reported among individuals with SSD [33, 34], our sample showed moderate adherence to general guidelines. Fruit and vegetable intake remained a potential area for improvement, even though it approached general recommendations [35, 36]. Meat consumption aligned with recommendations [35] and may reflect the lower socioeconomic status common among individuals with SSD [37, 38]. Candy/soda consumption aligned with sugar-intake norms [36]. Consistent with these findings, prior research found no direct differences in dietary habits between individuals with schizophrenia and matched controls, apart from lower fruit and vegetable consumption [34, 39, 40].

After the intervention, dietary indicators did not improve and shifted toward lower fruit and vegetable intake and higher candy/soda consumption. This contrasts with general-population studies suggesting that physical activity can improve appetite control and reduce cravings for unhealthy foods [41, 42]. Thus, individuals with SSD may require additional targeted nutritional support to change dietary habits.

The observed decrease in BMI deviates from the typical trajectory in this patient group, where obesity is common and antipsychotics often lead to weight gain [43]. However, the BMI decrease was not associated with any of the studied lifestyle habits but may reflect increased energy expenditure or other unmeasured factors.

### Limitations

This study used data from the FitForLife project, a single-arm intervention without a control group. While this design ethically allowed all eligible individuals to participate and avoided withholding a potentially beneficial exercise program [44], it limits the conclusions [45]. Additionally, the six-month timeframe may have been too short to detect long-term behavioral changes, especially in entrenched habits such as nicotine use.

Recruiting 149 participants with SSD—of whom 44 did not complete follow-up—was reasonably robust given the challenges of engaging and retaining individuals with severe mental illnesses [46, 47]. This was reflected in few significant results suggesting a need for a larger sample. Notably, 52% of the participants were men, contributing to the representativeness of the sample, as men are generally underrepresented in studies [48] and often experience more severe symptoms [49].

In terms of potential selection bias, individuals who opted into the intervention may differ from those who declined. Logistic regression showed no significant differences in measured covariates between completers and non-completers, but unmeasured factors may still vary, so selection bias cannot be fully excluded.

The dietary questions were chosen from nine questions originally designed for a microbiota study; four were used in the current study. These four were selected in collaboration with public health and nutrition researchers and broadly aligned with dietary recommendations [35]. As these dietary items were not tailored specifically for the current study, future research should consider more precise or extensive dietary measures, including fiber intake and high-fat foods. Another limitation of the dietary questions is the absence of gram-based response categories, which hinders direct comparison with general guidelines.

Response rates varied, with some categories receiving few responses, possibly reflecting discomfort or fear of repercussions, especially regarding sensitive behaviors like nicotine or alcohol use. Although questionnaires were anonymous, administration within treatment settings may have led to underreporting. Stigma remains a critical barrier in this population, potentially deterring honest reporting or proactive behavior change [50]. Addressing stigma and fear of repercussions may improve response rates and reporting accuracy in future interventions.

In summary, this study suggests that physical exercise interventions alone may be insufficient to change other lifestyle habits in individuals with SSD. This has implications for how interventions/treatment strategies are designed. Future research should explore larger/longer studies to determine if the observed patterns persist and to identify if integrated programs could improve lifestyle habits and thereafter narrow the life-expectancy gap and reduce long-term healthcare costs.

## Supplementary Information

Below is the link to the electronic supplementary material.

Supplementary Material 1.

Supplementary Material 2.

Supplementary Material 3.

## Data Availability

The datasets used and analyzed during the current study are available from the corresponding author on reasonable request.

## Abbreviations

AUDIT: Alcohol use disorders identification test
BMI: Body mass index
CI: Confidence intervals
OR: Odds ratio
SSD: Schizophrenia spectrum disorders
WHO: World Health Organization
