# Supplementary figures and images for: The impact of a national exercise intervention on lifestyle habits among individuals with schizophrenia spectrum disorders: results from the FitForLife intervention study
Source: BMC Res Notes. 2026 Apr 15;19:187. doi: 10.1186/s13104-026-07801-x (PMC13123093; doi:10.1186/s13104-026-07801-x)

**Figure 1. Flow chart**

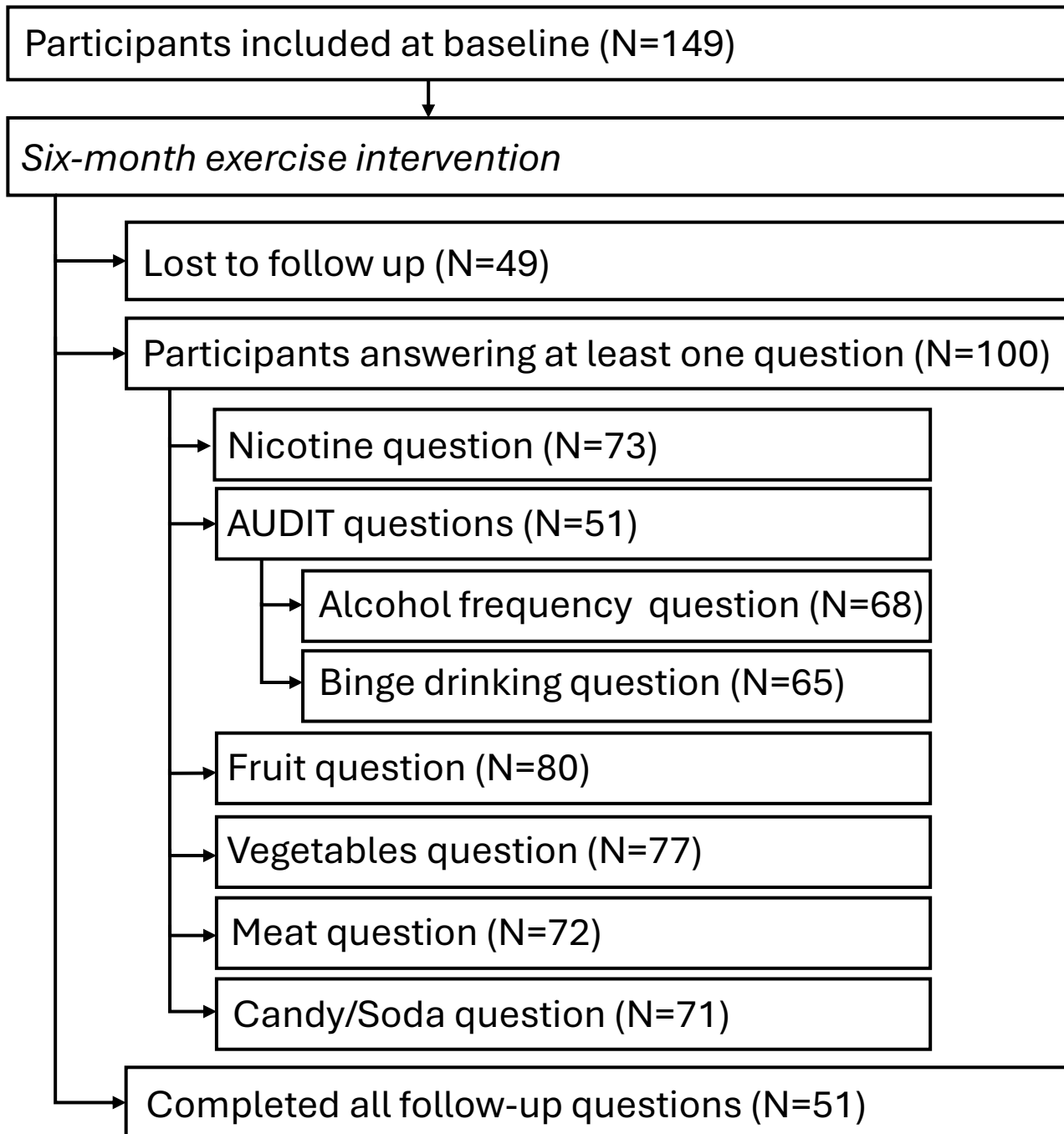

Supplement: Supplementary file 1 — Supplementary Material 1. [file 13104_2026_7801_MOESM1_ESM.pdf]
