# Supplementary material for: The impact of a national exercise intervention on lifestyle habits among individuals with schizophrenia spectrum disorders: results from the FitForLife intervention study
Source: BMC Res Notes. 2026 Apr 15;19:187. doi: 10.1186/s13104-026-07801-x (PMC13123093; doi:10.1186/s13104-026-07801-x)
Supplement: Supplementary file 3 — Supplementary Material 3. [file 13104_2026_7801_MOESM3_ESM.docx]

# Questionnaire FitForLife: Questions regarding study on lifestyle habits

*Translated from Swedish*

Living conditions/arrangements

Q42 Do you live together with another adult?

*Answer:* “no | “yes, permanently” | “yes, parent” | “yes, other”

Q43 Highest achieved education?

*Answer:* **1**= ”General education” (Grundskola/enhetsskola), **2**= ”High school”, practical (Fackskola, yrkesskola, gymnasieskola), **3**= ”high school, theoretical” (Gymnasieskola teoretiska linjer), **4**= ”higher education” (eftergymnasial utbilding, minst två år).

Tobacco

Q53 Have you used any tobacco products (Ex. Cigarettes, e-cigarettes, snus) during the past month?

*Answer:* no | yes

Alcohol

Q60 How often do you drink alcohol?

Answer: “never” (skip to the next question) | “1 time per month or less” | “2-4 times per month” | “2-3 times per week” | “4 times per week or more”

Q61 How many alcoholic units do you drink on a typical day when you drink alcohol?


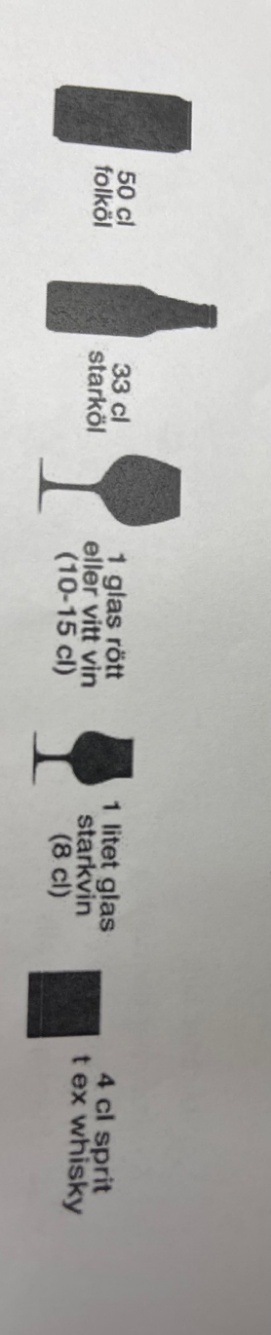


Answer: “1-2” | “3-4” | “5-6” | “7-9” | “10 or more”

Q62 How often do you drink six or more units during the same day?

*Answer:* 1= never, 2= less than 1 time per month, 3= every month, 4= every week, 5= Every day or almost every day

Q63 during the past year, how often have you not been able to stop drinking after you have started?

*Answer:* 1= never, 2= less than 1 time per month, 3= every month, 4= every week, 5= Every day or almost every day

Q65 during the past year, how often have you not done something you had to do because of your drinking?

*Answer:* 1= never, 2= less than 1 time per month, 3= every month, 4= every week, 5= Every day or almost every day

Q66 during the past year, how often have you need a drink the morning after drinking a lot the day before?

*Answer:* 1= never, 2= less than 1 time per month, 3= every month, 4= every week, 5= Every day or almost every day

Q67 during the past year, how often have you felt guilty about something you said or did when you were drinking?

*Answer:* 1= never, 2= less than 1 time per month, 3= every month, 4= every week, 5= Every day or almost every day

Q68 during the past year, how often have you not remembered what you said or did because of drinking?

*Answer:* 1= never, 2= less than 1 time per month, 3= every month, 4= every week, 5= Every day or almost every day

Q69 Have you or someone else ever been physically hurt because of your drinking?

*Answer:* “No” | “yes, but not during the past year” | “yes, recently”

Q70 Have a friend, family member, a doctor or someone else working within health care worried about your alcohol consumption and expressed that you should reduce your drinking?

*Answer:* “No” | “yes, but not during the past year” | “yes, recently”

Narcotics

Q71 Have you smoked /used cannabis during the past month?

Answer: “No” (skip to the next section) | “Yes”

Considering the past week, how many times did you eat/drink the following?:

Q83 Fruit

*Answer:* “1-2” | “3-4” | “5-6” | “6+”

Q84 Vegetables

*Answer:* “1-2” | “3-4” | “5-6” | “6+”

Q88 Meat

*Answer:* “1-2” | “3-4” | “5-6” | “6+”

Q91 Candy/ Soda

*Answer:* “1-2” | “3-4” | “5-6” | “6+”
